# Evaluation of Roasting and Grilling Effects on Chemical Composition, Volatile Profiles, and Toxicity of Stink Bugs (*Tessaratoma papillosa*): Implications for Utilization as Functional Food Ingredients

**DOI:** 10.3390/foods12163053

**Published:** 2023-08-15

**Authors:** Hua Li, Theeraphan Chumroenphat, Parinya Boonarsa, Jantana Yahuafai, Colin Wrigley, Sirithon Siriamornpun

**Affiliations:** 1 Department of Cuisine and Nutrition, Yangzhou University, Yangzhou 225127, China; 2 Research Unit of Thai Food Innovation (TFI), Mahasarakham University, Kantarawichai, Maha Sarakham 44150, Thailand; 3 Aesthetic Sciences and Health Program, Faculty of Thai Traditional and Alternative Medicine, Ubon Ratchathani Rajabhat University, Ubon Ratchathani 34000, Thailand; 4 Department of Food Technology and Nutrition, Faculty of Technology, Mahasarakham University, Kantarawichai, Maha Sarakham 44150, Thailand; 5 Clinical Research Section, Division of Research and Academic Support, National Cancer Institute, Bangkok 10400, Thailand; 6 Center for Crop Science, Queensland Alliance for Agriculture and Food Innovation, University of Queensland, Brisbane 4067, Australia

**Keywords:** edible insect, protein, amino acid, phenolic acid, tocopherol, anti-proliferative

## Abstract

The stink bug (*Tessaratoma papillosa*) is a highly popular edible insect in Thai traditional cuisine, but little research has investigated the effects of heat treatment on the quality of stink bugs. Therefore, we aimed to evaluate the effects of roasting and grilling on the chemical changes and volatile compounds of late nymph and adult stink bugs. In general, all treated samples showed increases in phenolic acid, tocopherols, and amino acid contents and a decrease in the content of fiber compared with raw stink bugs (*p* < 0.05). Cinnamic acid significantly increased by over 200% in late nymph insects and 30% in adult insects after roasting, whereas syringic acid decreased after cooking (*p* < 0.05). The most predominant volatile compound found in all samples was 5-methyl-octadecane and it decreased after cooking, while volatile alkane compounds increased after cooking. The processed sample extracts showed higher toxicity on oral cancer KB and cervical cancer Hela cells than on Vero cells. We have demonstrated that different cooking methods affected the chemical components which may result in quality attributes if stink bug is to be used as a functional ingredient/food. It may be helpful to improve the nutritional and functional values of stink bugs during deep processing.

## 1. Introduction

Insects have gained significant popularity as a food source among various population groups. Indigenous communities, particularly in Africa, Asia, and Latin America, including Thailand, have traditionally consumed a diverse range of insect species [1]. In fact, insects comprise more than 90% of the animal population [2]. These creatures possess exceptional nutritional value, characterized by relatively high protein and fat contents with favorable amino acid and fatty acid profiles, along with a rich array of micronutrients such as vitamins and minerals [3]. As a sustainable and efficient food source, insects have emerged as a viable alternative to conventional options [2]. Currently, ants, bees, wasps (Hymenoptera), beetles (Coleoptera), grasshoppers, locusts (Orthoptera), termites (Isoptera), true bugs, aphids, leafhoppers (Hemiptera), caterpillars (Lepidoptera), and flies (Diptera) are the prominent species consumed, encompassing various life stages, including eggs, larvae, pupae, and adults [4]. The stink bug (*Tessaratoma papillosa*) has emerged as a highly popular edible insect, particularly in the rural regions of Thailand, extending its recognition nationwide [5]. In Thai traditional or local cuisine, it is widely appreciated for its distinct pungent flavor, which complements dishes that call for unique and robust taste profiles, often achieved through the use of chili paste or a variety of dipping condiments [6].

According to several reviews [7,8,9], many studies have determined the nutritional composition and content of bioactive compounds of edible insects, but there is only a little research on the quality attributes of *T. papillosa* as food. For example, Raksakantong et al. [6] analyzed the fatty acid and proximate compositions of adult *T. papillosa*. Understanding the chemical composition of *T. papillosa* is crucial for identifying pheromones, so previous research has also focused on identifying volatile components that serve as important pheromones in adult *T. papillosa* [10]. This edible insect is consumed in various forms, including dried, grilled, roasted, deep-fat fried, stewed, microwave heated, boiled, and as a side dish or snack accompanying starchy meals [11]. Cooking processes not only enhance sensory quality, food safety, and shelf life, but also impact the nutritional content of edible insects [11]. Ssepuuya et al. [12] reported significant decreases in vitamin B12 and essential fatty acids in *Ruspolia differens* following roasting and boiling. Meanwhile, the chemical profiles of a given insect can vary with the stage of development [7].

However, limited information exists regarding the volatile, phenolic, and flavonoid components of *T. papillosa,* specifically during the late nymph and adult stages, and investigations into the volatile and phenolic components after cooking have not been previously explored. Therefore, this study aimed to evaluate and compare the content and composition of these compounds in *T. papillosa* at different instars using roasting and grilling techniques. Furthermore, the investigation encompassed proximate analysis, γ-oryzanol, α-, δ-, and γ-tocopherols, as well as amino acid contents to provide a comprehensive understanding of the chemical components. We hypothesized that the cooking methods employed prior to consumption may significantly influence the quality properties and nutritional value of cooked *T. papillosa*, and there may be a significant difference in some of these indexes of stink bugs at different developing stages.

## 2. Materials and Methods

### 2.1. Chemicals, Cells, and Cultures

We purchased all standards from Sigma-Aldrich Co. (St. Louis, MO, USA) for our analysis. The set of phenolic acid standards included gallic acid, protocatechuic acid, chlorogenic acid, *p*-hydroxybenzoic acid, vanillic acid, caffeic acid, vanillin, *p*-coumaric acid, ferulic acid, syringic acid, sinapic acid, cinnamic acid, and gentisic acid. Additionally, we utilized five flavonoid standards: rutin, quercetin, apigenin, kaempferol, and myricetin. For amino acid determination, we employed twenty standard compounds (arginine, histidine, isoleucine, leucine, lysine, methionine, phenylalanine, threonine, tryptophan, valine, alanine, asparagine, aspartic acid, cysteine, glutamine, glutamic acid, glycine, proline, serine, and tyrosine). The standards of Tocopherol (δ-tocopherol, γ-tocopherol, and α-tocopherol) and γ-oryzanol were also used. The chemicals of analytical grade included *n*-hexane, acetone, and hydrochloric acid. The solvent of chromatography analysis involved acetonitrile, methanol, and formic acid as HPLC grade.

The Human oral epithelial carcinoma cell line (KB), Human cervical cancer cell line (Hela), and normal Vero cell line (African Green Monkey Kidney) were acquired from ATCC. Vero and KB cell lines were cultured in Eagle’s minimal essential medium, while Hela cells were cultured in Dulbecco’s modified Eagle’s medium. The media were supplemented with 1% (*v*/*v*) antibiotic and 10% (*v*/*v*) fetal bovine serum, and the cell cultures were maintained in a CO_2_ incubator at 37 °C.

### 2.2. Sample Preparation

*T. papillosa*, at different growth stages, was obtained from the local market in Sakon Nakhon Province, Thailand. After fasting for 48 h, the stink bugs were roasted at 160 °C for 45 min with an electric pan (GR 141, OTTO, Bangkok, Thailand) or grilled at 160 °C for 5 min on an electric stove (JMS-27, Jiahuang, China) using thermometer TTX 100. The samples were then ground and stored at −20 °C until used.

### 2.3. Determination of Proximate Composition

According to AOAC official method (2000), protein (981.10), fat (960.39), fiber (934.01), and ash (920.153) contents were measured. The carbohydrate content was calculated as [100 − (%protein + %fat + %fiber + %ash)].

### 2.4. HPLC Determination of Phenolic Acids and Flavonoids

Phenolic acids and flavonoids were extracted following the method of Chumroenphat et al. [13] and were analyzed using the HPLC method [14]. The condition of HPLC (series 20, Shimadzu, Kyoto, Japan) was as follows: C18 column (250 mm × 4.6 mm, 5 µm; InertSustain, GL Sciences, Tokyo, Japan); the mobile phase consisted of purified water with 1% (*v*/*v*) acetic acid (solvent A) and acetonitrile (solvent B) at a flow rate of 0.8 mL/min. A linear gradient elution was performed as follows: 0–5 min, 5–9% B; 5–15 min, 9% B; 15–22 min, 9–11% B; 22–38 min, 11–18% B; 38–43 min, 18–23% B; 43–44 min, 23–90% B; 44–45 min, 90–80% B; 45–55 min, 80% B; 55–60 min, 80–5% B. A re-equilibration period of 5 min with 5% B was used between individual runs. Operating conditions were as follows: column temperature, 38 °C; injection volume, 20 µL; detection wavelength including 280 nm (hydroxybenzoic acids), 320 nm (hydroxycinnamic acids), and 370 nm (flavonoids). The phenolic compounds in the extracts were identified using the method of external standards. Linear equations for the quantitative calculation of concentration are shown in Table S1.

### 2.5. Extraction and Determination of γ-Oryzanol and α-, δ-, and γ-Tocopherols

The extraction of γ-oryzanol and tocopherols followed the method described by Chen and Bergman [15] with slight modifications. A stink bug sample weighing one gram was vortexed with a mixture of acetone and water (1:10, *v*/*v*), followed by centrifugation (Sigma 6–16K benchtop centrifuge, Sigma-Aldrich, Castle Hill, NSW, Australia) at 2500 rpm for 20 min. The process of extraction was repeated twice using the same procedure. The resulting supernatants were combined, and the solvent was evaporated under a stream of nitrogen gas until dryness was achieved. Subsequently, the extracts were subjected to separation of γ-oryzanol and tocopherols using a reverse-phase high-performance liquid chromatography (RP-HPLC) system (series 20, Shimadzu, Kyoto, Japan) with C18 column (250 mm × 4.6 mm, 5 µm; InertSustain, GL Sciences, Tokyo, Japan). The mobile phase employed was acetonitrile/methanol (25:75, *v*/*v*), and isocratic elution was carried out at a flow rate of 1.5 mL/min. The wavelengths of 292 nm and 325 nm were set for the analysis of tocopherols and γ-oryzanol, respectively. Calibration curves were constructed with external standards. Linear equations for the quantitative calculation of concentration are shown in Table S1.

### 2.6. Determination of Amino Acid Content

The *T. papillosa* samples were procured from food markets in Sakon Nakhon Province, Thailand, and subsequently subjected to freeze-drying prior to analysis. To obtain the defatted sample, 5 g of the ground freeze-dried powder of *T. papillosa* was dispersed in 50 mL of *n*-hexane and sonicated for 30 min. The residue was extracted three times with *n*-hexane. After removing the residual hexane, the defatted sample underwent hydrolysis by adding 5 mL of 6 mol/L HC1 and incubating it at 110 °C in a hot-air oven (FED 115, WTB Binder, Tuttlingen, Germany) for 15 h. The HC1 was then evaporated (R-210, BUCHI Labortechnik AG, Flawil, Switzerland) at 60 °C. The resulting dried hydrolysate was dissolved in a tenfold volume of water and centrifuged (Sigma 6–16K benchtop centrifuge, Sigma-Aldrich, Castle Hill, NSW, Australia) at 12,000× *g* for 10 min. After filtration using a 0.22 µm nylon membrane, the supernatant was subjected to LC/MS/MS analysis (LCMS-8030, Shimadzu, Kyoto, Japan) using a C18 column (150 mm × 2.1 mm, 3 µm; InertSustain, GL Sciences, Tokyo, Japan) for amino acid separation. The LC/MS/MS conditions were set following the method described by Chumroenphat et al. [13]. The mobile phases used were formic acid 0.1% (*v*/*v*) in water (solvent A) and formic acid 0.1% (*v*/*v*) in water/methanol (50:50, *v*/*v*) (solvent B). A linear gradient elution was performed as follows: 2% B (0–1 min), 2–80% B (1–10 min), 80% B (10–12 min), and 80–2% B (12–15 min). The flow rate was 0.2 mL/min, and the injection volume was 2 μL. The column oven and auto injection temperature were 38 °C and 15 °C, respectively. The triple quadrupole mass spectrometer was used with an electrospray ionization ion source in positive mode and multiple reaction monitoring. Other MS parameters were as follows: interface 4.5 kV, interface temperature 250 °C, desolvation line 300 °C, heat block 350 °C, detector 1.78 kV, nebulizing gas (N_2_) 3 L/min, drying gas (N_2_) 15 L/min, collision with argon gas at 230 kPa. The results were expressed as milligrams of amino acids per gram on a dry basis. The essential amino acid index (EAAI) was calculated using the method outlined by Yi et al. [4]. Linear equations for the quantitative calculation of concentration are shown in Table S1.

$$\mathrm{EAAI}=\sqrt[{9}]{\frac{\mathrm{mg}\;\mathrm{of}\;\mathrm{lysine}\;\mathrm{in}\;1\;g\;\mathrm{of}\;\mathrm{test}\;\mathrm{protein}}{\mathrm{mg}\;\mathrm{of}\;\mathrm{lysine}\;\mathrm{in}\;1\;g\;\mathrm{of}\;\mathrm{reference}\;\mathrm{protein}}\times etc.\;for\;the\;other\;8\;essential\;amino\;acids}$$

### 2.7. Determination of Volatile Compounds

The extraction of volatile constituents from the samples was performed using HS-SPME (headspace solid-phase microextraction), following the method outlined by Zhang et al. [16] with some modifications. The samples, weighing 2.0 g, were subjected to extraction of volatiles for 30 min using a divinylbenzene/carboxin/polydimethylsiloxane (DVB/CAR/PDMS/10, Agilent Technologies, Palo Alto, CA, USA) fiber, which had been prepared at 250 °C for 5 min. Subsequently, the SPME fiber was desorbed at 250 °C for 5 min to concentrate the analytes.

The volatile components present in the stink bug samples were analyzed using a GC/MS system (QP2010, Shimadzu, Tokyo, Japan) equipped with a fused silica capillary column (30 m × 0.25 mm, 0.25 µm; Rtx-5MS, Restek Co., Bellefonte, PA, USA). The column temperature and MS conditions followed the method described by Zhang et al. [16]. The oven temperature of the GC program was as follows: 50 °C for 5 min, increasing to 100 °C (3 °C/min), holding for 5 min, increasing to 250 °C (5 °C/min), holding for 3 min, and a final ramp to 280 °C (50 °C/min), holding for 5 min. The MS was operated in the full-scan range (*m*/*z* 40–550 amu) with electron ionization mode (70 eV). The identification of peaks was accomplished by comparing the mass fragmentation pattern of each compound with data available in the NIST14 mass spectral library. The relative content of each compound was expressed as a percentage of its peak area in relation to the total peak area.

### 2.8. Determination of Molecular Weights of Protein Components

#### 2.8.1. Protein Extraction Procedure

The ground freeze-dried insect powder was defatted as described in part 2.6, and then 1 g of the defatted powder was mixed with 10 mL of 0.02% (m/v) ascorbic acid and homogenized. After filtering through medical gauze, the filtrate was centrifuged (Sigma 6–16K benchtop centrifuge, Sigma-Aldrich, Castle Hill, NSW, Australia) at 10,000× *g* for 30 min. The supernatant was collected for the Western analysis.

#### 2.8.2. Evaluation of Protein Molecular Weight

Protein molecular weight was determined using capillary-based western analysis, following the protocol of the Protein Simple Jess system (Jess, Protein Simple, CA, USA). In this method, the cell lysates were diluted in a sample buffer to a concentration of 1 mg/mL. A master mix containing 40 mmol/L dithiothreitol, 1× sample buffer, and 1× fluorescent standard was thoroughly mixed with each sample. The mixture was then heated at 95 °C for 5 min to denature the proteins. Subsequently, 3 μL of the denatured protein extract and 10 μL of each primary antibody, diluted at a ratio of 1:20, along with HRP-conjugated anti-mouse secondary antibodies, protein normalization solution, and chemiluminescent substrate, were loaded. The primary antibodies used in this study were Anti-JAK2 (NBP2-59451; Novus Biologicals, LLC, Littleton, CO, USA) and anti-STAT5 (AF2168; R&D System, Minneapolis, MN, USA). To ensure accurate protein loading, total protein was normalized within the capillaries. For each assay, standards with molecular weights ranging from 12 to 230 kDa were employed. The chemiluminescent reactions were analyzed using Compass software (Protein Simple).

### 2.9. Cell Proliferation Assay

To obtain the extracts for the cell proliferation assay, the following procedures were conducted. A mixture comprising 1 g of freeze-dried stink bug powder and 10 mL of distilled water was subjected to sonication at room temperature for 30 min (cold extraction) or heated in a water bath at 90 °C for 15 min (heat extraction), followed by filtration. The resulting supernatants were subsequently freeze-dried.

The cytotoxic effect of the stink bugs was evaluated using the MTT assay, following the previously described method by Siripong et al. [17]. In brief, cells were incubated in a 96-well plate at a density of 3 × 10^3^ cells per well for 24 h. After the pre-incubation period, the cells were exposed to serial concentrations of the aqueous extract (ranging from 31.25 to 2000 µg/mL) for 72 h. Doxorubicin (at concentrations of 0.01 to 10 µg/mL) served as the positive control. Subsequently, 20 µL of a 5 mg/mL MTT solution in PBS was added, followed by further incubation at 37 °C for 3 h. After removing the medium, the formazan crystals were dissolved in DMSO. The absorbance at 550 nm was measured using a Microplate reader (Benchmark 550, Bio-Rad, Hercules, CA, USA). The IC_50_ value was calculated by plotting the percentage of cell viability against the concentrations of the extract or control.

### 2.10. Statistical Analysis

All results are expressed as mean ± standard deviation of three replicates. The significant difference was evaluated by the least significant difference test using one-way ANOVA in SPSS statistical software (version 16.0, SPSS Inc., Chicago, IL, USA). Different letters among samples or parameters indicate significant differences (*p* < 0.05).

## 3. Results

### 3.1. General Characteristics of Stink Bugs

**Figure 1:**
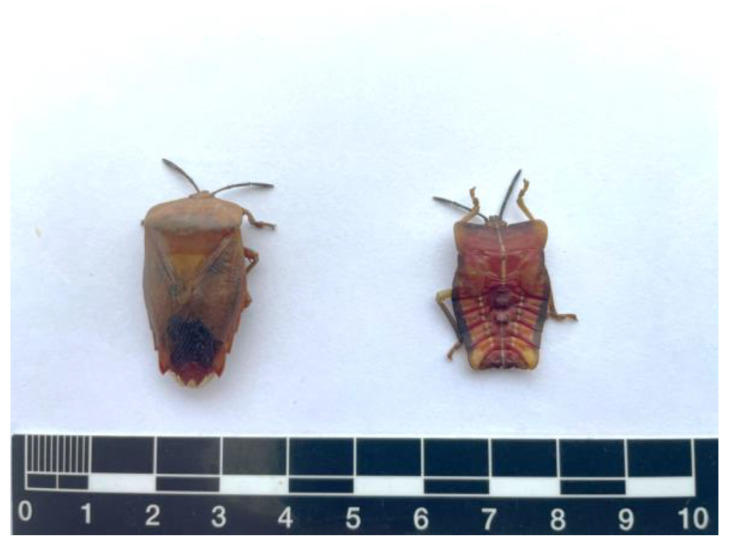
Stink bugs at different growth stages, namely late nymph and adult.

Stink bugs, representing various growth stages, namely late nymphs and adults, were meticulously collected from local areas (Figure 1). Prior to analysis, both the late nymph and adult samples underwent freeze-drying. The characteristics of the stink bugs, including size, morphology, and local cooking styles, are comprehensively presented in Table 1.

### 3.2. Phenolic Acid and Flavonoid Contents

Table 2 provides an overview of the total phenolic acid and flavonoid contents in *T. papillosa* cooked using different methods. The results revealed higher total phenolic acid levels in adults, while late nymph samples exhibited higher total flavonoid levels. Syringic acid and cinnamic acid were identified as the major phenolic compounds in both sample types, with vanillic acid exclusively present in late nymphs. The roasting and grilling treatments significantly enhanced the total phenolic acid and flavonoid contents in both sample types. Remarkably, the content of cinnamic acid increased significantly by 243% in late nymphs and by 30% in adults after roasting. In contrast, the content of syringic acid decreased substantially following roasting and grilling. These findings align with previous studies on other food sources, such as *Arachis hypogaea* L. [20] and selected nuts and oilseeds [21], which reported increased the phenolic content due to the roasting process.

Regarding flavonoids, kaempferol was exclusively found in late nymphs, while myricetin emerged as the predominant compound, with concentrations of 169 μg/g in late nymphs and 80 μg/g in adults. The roasting process led to a substantial increase of 343% and 53% in the myricetin content in late nymphs and adults, respectively. These observations align with previous reports on roasted peanuts, which demonstrated a significant increase in the flavonoid content compared to raw peanuts [20,22]. The improvements observed in the total phenolic acid and flavonoid contents of *T. papillosa*, particularly after roasting, can be attributed to the breakdown of cellular constituents, resulting in the release of free phenolic and flavonoid compounds, as well as the formation of heat-induced and extractable phenolic and flavonoid compounds [23].

Based on these findings, roasting appears to be a suitable cooking method for *T. papillosa*, as it enhances the phenolic acid and flavonoid contents, which serve as valuable sources of cinnamic acid and myricetin. These components have been associated with numerous potential health benefits, including anti-inflammatory properties, blood sugar and cholesterol level regulation, memory improvement, and promotion of beneficial gut bacteria growth [24].

### 3.3. Oryzanol and Tocopherols Contents

In this study, we aimed to investigate the contents of oryzanol and tocopherols in roasted and grilled *T. papillosa* (Table 3). Our findings revealed that γ-tocopherol was the most abundant tocopherol in raw *T. papillosa*, followed by α-tocopherol and δ-tocopherol. However, γ-oryzanol was not detected in any of the samples. Interestingly, the amount of tocopherols showed significant changes based on the cooking method employed. Roasting led to a significant increase in the tocopherol content, while grilling resulted in a decrease. This could be attributed to the higher temperatures involved in grilling, which may have caused an inverse effect [25].

Specifically, the amount of α-tocopherol increased significantly by 264% in late nymph samples after roasting, while the γ-tocopherol content increased significantly by 319% in adult samples. Jannat et al. [25] reported a similar increase in the γ-tocopherol content in Iranian sesame seeds with rising roasting temperature and time, up to 200 °C. Furthermore, Silva et al. [26] found no significant differences in the tocopherol contents between raw and roasted peanuts, except for a decrease in the α-tocopherol content after roasting. Based on our results, late nymphs and adults of *T. papillosa* can be considered as valuable sources of γ-tocopherol, both for use as supplements and as food, especially after the roasting process. These findings contribute to our understanding of the potential nutritional benefits of *T. papillosa* and its utilization in various applications.

### 3.4. Proximate Composition

The proximate compositions of *T. papillosa* following various cooking methods are summarized in Table 3. Our results indicate that the cooked insects did not exhibit significant differences in the fat content compared to the raw samples. On a dry-weight basis, late nymphs displayed a higher protein content, whereas adults exhibited a higher fat concentration. Notably, there were no significant differences in the protein content between the cooked late nymph samples and the raw samples, while roasting led to a significant increase in the protein content for the adult samples. This aligns with the findings of Oluwaniyi et al. [27], who reported an increase in protein content for *Clarias gariepinus* and *Oreochromis niloticus* following the roasting process.

Regarding the fiber content, both the roasting and grilling methods had a significant impact on the edible insects. The fiber content of late nymphs followed the decreasing order of raw (14.2%) > roasted (12.7%) > grilled (12.5%). Similarly, the fiber content of adult samples followed the decreasing order of raw (14.3%) > roasted (12.1%) > grilled (11.7%). The reduction in the fiber content in the roasted and grilled samples could be attributed to the loss of certain chemical components, such as cellulose, pectic substances, hemicellulose, mucilage, gums, or lignin, or the alteration of the structural functionality of the fiber content within the samples [28]. This is consistent with the findings of Thomas et al. [29], who reported depletion of cellulose, hemicellulose, and lignin in a variety of cooked potato tubers under different conditions.

The ash content of late nymphs significantly increased by 50.5% after roasting, while the grilling method exhibited a significant change in adult samples with an increase of 30.9%. These increases in the ash content during roasting and grilling indicate an enhancement in the mineral content of *T. papillosa* [28]. Similarly, Cano-Estrada et al. [30] reported an increase in the ash content of rainbow trout during grilling. The carbohydrate content was determined to assess the total energy value of this edible insect. The grilled method significantly increased the percentage of carbohydrates in *T. papillosa*, indicating that the insect can serve as a good source of energy for humans.

### 3.5. Amino Acid Content

The total amino acid content and composition of twenty amino acids in raw and cooked *T. papillosa* are presented in Table 4. Our results indicate that late nymphs had a higher total amino acid content compared to adults. Furthermore, the roasting process significantly increased the amino acid content of *T. papillosa*, while the grilling process led to a slight increase in the total amino acid content. Hence, the roasting method proved to be more effective than grilling in enhancing the amino acid content. Among the amino acids, tyrosine (70 mg/g) was the most abundant in late nymphs, followed by methionine and histidine (53 and 25 mg/g, respectively). Conversely, methionine was the most abundant amino acid in adults, followed by tyrosine and histidine (46 and 19 mg/g, respectively). No presence of asparagine was detected in any of the samples.

The roasting treatment exhibited a tendency to increase the free amino acid content in late nymphs. Specifically, the levels of arginine, isoleucine, leucine, and tyrosine increased by approximately 4.48%, 4.52%, 6.43%, and 5.83%, respectively, compared to the raw samples. Similarly, in adults, methionine, glutamic acid, leucine, and aspartic acid increased by approximately 8.45%, 12.06%, 8.30%, and 11.89%, respectively, after roasting. These findings are consistent with the results of Adu et al. [31], who reported that roasting treatment in *Terminalia catappa* significantly improved the availability of the total amino acid content by up to 11%. Additionally, Lopes et al. [32] observed a significant increase in the total amino acid content of Barrosã-PDO veal after grilling.

These findings highlight the impact of cooking methods, particularly roasting, on the amino acid content and composition of *T. papillosa*. The enhanced amino acid profiles contribute to the nutritional value of this edible insect, making it a potential source of essential amino acids in human diets.

### 3.6. Volatile Compounds Content

The impact of different cooking methods on the volatile compounds extracted from raw and cooked *T. papillosa* is presented in Table 5. A comprehensive analysis using the SPME-GC-MS method revealed a total of 23 volatile compounds. Notably, 5-methyl-octadecane emerged as the predominant compound in both late nymphs (88.59%) and adults (92.24%). This prominent alkane is also found in various plant species and has exhibited diverse pharmacological effects. These plants hold promise as potential sources of oils that could provide antioxidant and antimicrobial compounds [33]. These findings align with Wang et al. [10], who reported that certain compounds were common to both nymphs and adult *T. papillosa*, with alkanes being notable components at high levels.

Moreover, specific volatile compounds such as 2-octenoic acid, *cis*-10-heptadecenoic acid, 10(E), 12(Z)-conjugated linoleic acid, 9-tricosanol, acetate, and hexatriacontane were exclusively detected in raw late nymphs, while dimethyl trisulfide, 2-hexenoic acid, and undecane were exclusively found in adult samples. However, 2-octenoic acid and *cis*-10-heptadecenoic acid were no longer detected after roasting and grilling, which can be attributed to the breakdown of cellular constituents at high temperatures [23]. Interestingly, alkane contents tended to be higher after roasting compared to grilling. Alkanes constituted the major chemical family in *T. papillosa* after roasting, accounting for approximately 90% and 95% of the total volatile compounds in late nymphs and adults, respectively. Conversely, the content of carboxylic compounds significantly increased in grilled samples, suggesting greater lipid degradation compared to roasted samples. These observations are consistent with previous findings by Domínguez et al. [34].

The aroma and flavor characteristics of cooked food significantly influence consumer acceptance and preference. Van et al. [35] explained that the main sources of volatile compounds in cooked meat are the thermal degradation of lipids, the Maillard reaction, interactions between Maillard-reaction products and lipid-oxidized products, as well as vitamin degradation.

### 3.7. Protein Molecular Weight in T. papillosa with Different Cooking Methods

**Figure 2:**
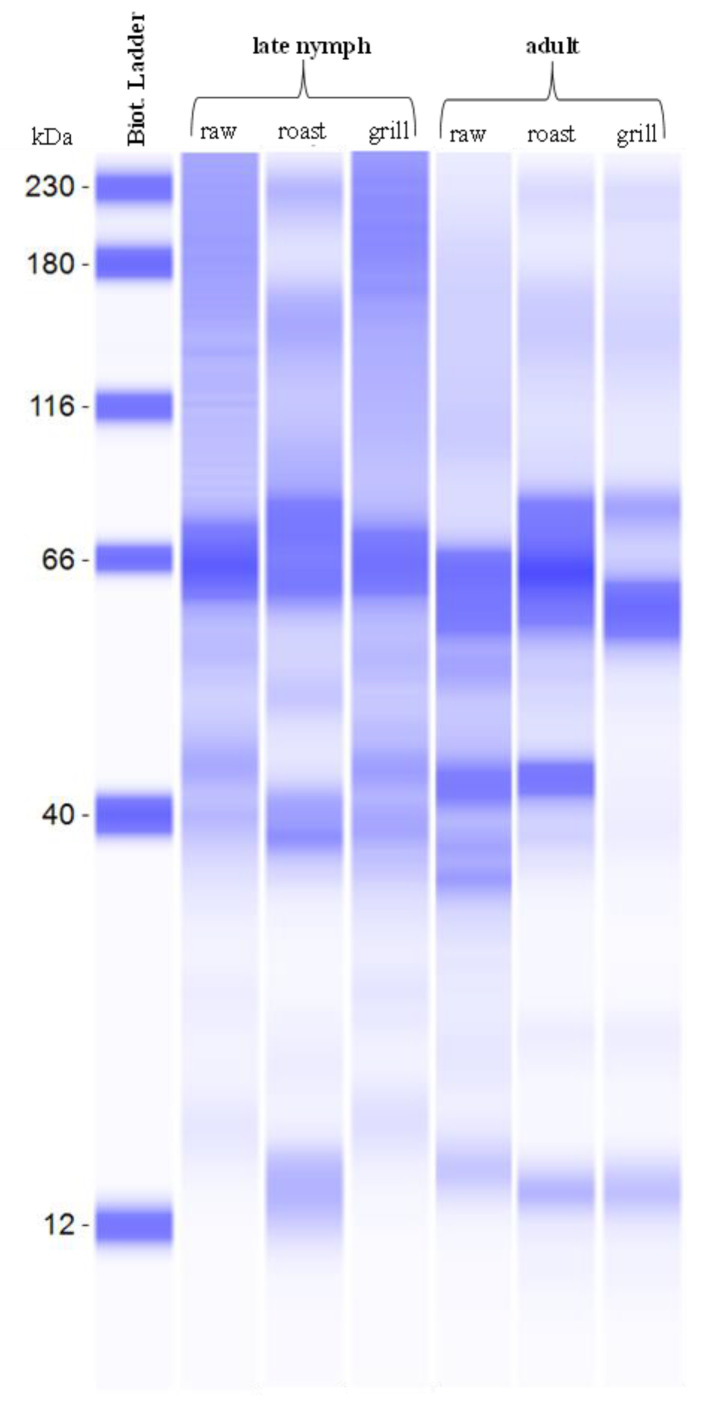
Molecular weight of proteins in stink bugs with different cooking methods.

The SDS-PAGE protein separations (Figure 2) reveal insights into the structural changes of raw, grilled, and roasted *T. papillosa*. The protein bands in the high-molecular-weight (HMW) range of 40–107 kDa were observed. Roasting had a significant impact on the protein structure, resulting in noticeable changes, while grilling showed slight alterations compared to the raw samples. This trend aligns with the changes observed in the amino acid contents of edible insects. For instance, Yu [36] demonstrated that the secondary structure of proteins in flaxseeds underwent significant transformations following roasting at 165 °C, leading to an increase in the β-sheet structure to α-helix structure ratio. Similarly, Waszkowiak and Mikołajczak [37] reported notable changes in the SDS-PAGE profiles of roasted seeds, particularly in protein fractions with molecular weights of 13 kDa (decrease), 19 kDa, and 17 kDa (increase), attributed to oxidation processes and the formation of Maillard reaction products.

In our study, some HMW proteins were observed to disappear after roasting. Manditsera et al. [38] explained that the protein profile of raw samples exhibited more similarity to that of roasted insects, distinct from that of boiled samples. A decrease in the number and intensity of bands in pellets was evident. However, boiling was not included in our present study as it is not a typical cooking method for stink bugs. The observed changes in the SDS-PAGE protein profiles may be attributed to structural modifications, including denaturation, aggregation, degradation, and cross-linking with polypeptides and other compounds, as seen in flaxseeds [37]. Therefore, the roasting treatment appears to offer enhanced functionality for *T. papillosa*, as evidenced by the wide dispersion of protein bands.

### 3.8. Anti-Proliferative Activity and Cytotoxicity of Stink Bug Extract

The half maximal inhibitory concentration (IC_50_) values of *T. papillosa* for Hela and KB cancer cells were compared to those for normal Vero cells, as presented in Table 6. Overall, the cytotoxicity test of *T. papillosa* extract revealed lower toxicity on Vero cells compared to Hela and KB cells. Intriguingly, the cooking process, specifically roasting and grilling, significantly enhanced the inhibition of Hela and KB cancer cells compared to fresh *T. papillosa*. For late nymphs, roasting exhibited the highest efficacy in inhibiting cancer cells, resulting in reduced IC_50_ values of 394.9 µg/mL in the Hela cells and 285.4 µg/mL in the KB cells. Conversely, for adults, the grilling method proved to be the most effective, leading to IC_50_ values of 257.7 µg/mL in the Hela cells and 303.1 µg/mL in the KB cells. These findings can be attributed to the presence of high total phenolic, tocopherol, and free amino acid contents, which are known to significantly reduce the survival of Hela and KB cells. The cell survival rate decreased by over 50% following the extraction of *T. papillosa*, suggesting that the roasting and grilling of *T. papillosa* are highly suitable for consumption. In addition to being an excellent protein source, it also exhibits promising potential in inhibiting cancer cells.

## 4. Conclusions

Based on the findings of this study, as we have hypothesized, the adult stink bug possessed considerably higher total phenolic acid and fat contents but lower total flavonoid and protein contents than the late nymph. Furthermore, the cooking methods of roasting and grilling exerted significant effects on the chemical composition and volatile compounds of both late nymphs and adults of the stink bug. Notably, these cooking methods led to substantial increases in phenolic acid, tocopherol, and amino acid contents. In late nymph samples, the total amino acid content remained high after roasting and grilling, while grilling showed a notable decrease in this factor for adult samples. The most prominent γ-tocopherol also exhibited a significant increase after roasting, reaching 1.35 times in late nymphs and 4.2 times in adults. Furthermore, the volatile compounds showed a tendency for higher alkane contents after roasting, accounting for approximately 90% in late nymphs and 95% in adults. Interestingly, 10(E), 12(Z)-conjugated linoleic acid was detected in fresh late nymph samples and its content tended to increase after roasting and grilling. These findings support the suitability of roasting and grilling as cooking methods for *T. papillosa* consumption, thereby enhancing its nutritional value. It is worth mentioning that *T. papillosa* also exhibits potential for inhibiting cancer cells, although further investigations with different concentrations or forms are necessary to explore this aspect.

## Figures and Tables

**Table 1:** Characteristics of stink bugs at different growth stages.

Samples	Size	Morphology	Local Style of Cooking	References
Late nymph	1.5–10.4 mm long	Yellow-brown in color Moderate pungent smell Wingless	Grilling Roasting Frying Light curry with vegetables	[6,18,19]
Adult	11.0–15.0 mm long	Brown marbled appearance Strong pungent smell Wing	Chili paste Dipping Grilling Roasting Frying	[6,18,19]

**Table 2:** Contents of phenolic acids and flavonoids in stink bugs with different cooking methods.

Parameter	Late Nymph			Adult		
	Raw	Roasted	Grilled	Raw	Roasted	Grilled
**Phenolic acid (μg/g)**						
gallic acid	158.8 ± 9.0 ^b^	381.4 ± 3.2 ^a^	155.7 ± 1.8 ^b^	98.0 ± 0.5 ^c^	109.8 ± 0.3 ^a^	107.6 ± 0.5 ^b^
protocatechuic acid	20.4 ± 0.5 ^b^	40.4 ± 4.4 ^a^	21.1 ± 0.5 ^b^	14.0 ± 0.1 ^b^	14.9 ± 0.7 ^a^	13.9 ± 0.3 ^b^
*p*-hydroxybenzoic acid	30.5 ± 0.6 ^c^	96.4 ± 4.9 ^a^	38.2 ± 0.4 ^b^	20.0 ± 0.0 ^b^	21.8 ± 0.1 ^a^	21.9 ± 0.1 ^a^
chlorogenic acid	-	-	-	-	-	-
vanillic acid	20.8 ± 0.2 ^c^	58.3 ± 0.5 ^a^	26.2 ± 0.2 ^b^	-	-	-
caffeic acid	-	-	1.5 ± 0.0 ^a^	-	-	-
syringic acid	266.4 ± 1.9 ^a^	-	171.1 ± 0.8 ^b^	121.8 ± 0.9 ^a^	60.3 ± 0.5 ^c^	71.5 ± 0.3 ^b^
vanillin	24.2 ± 1.2 ^c^	62.4 ± 0.4 ^a^	26.1 ± 0.3 ^b^	10.4 ± 0.1 ^a^	8.9 ± 0.2 ^b^	8.9 ± 0.1 ^b^
*p*-coumaric acid	-	-	1.0 ± 0.1 ^a^	-	-	-
ferulic acid	2.1 ± 0.2 ^b^	-	3.2 ± 0.1 ^a^	0.8 ± 0.0 ^a^	0.8 ± 0.0 ^a^	0.8 ± 0.0 ^a^
sinapic acid	0.7 ± 0.1 ^c^	7.1 ± 0.4 ^a^	1.2 ± 0.0 ^b^	0.6 ± 0.0 ^c^	0.7 ± 0.0 ^b^	0.8 ± 0.0 ^a^
cinamic acid	160.8 ± 8.9 ^c^	552.8 ± 10.6 ^a^	246.1 ± 4.5 ^b^	1438.6 ± 23.1 ^c^	1867.7 ± 23.3 ^a^	1733.0 ± 6.5 ^b^
genistic acid	18.1 ± 0.7 ^c^	39.1 ± 2.3 ^a^	29.1 ± 1.5 ^b^	147.8 ± 0.8 ^b^	153.9 ± 10.7 ^b^	180.2 ± 0.9 ^a^
total	702.8 ± 25.8 ^b^	1237.8 ± 25.3 ^a^	720.5 ± 10.2 ^b^	1852.0 ± 25.7 ^c^	2238.8 ± 35.9 ^a^	2138.6 ± 8.6 ^b^
**Flavonoid (μg/g)**						
rutin	29.7 ± 0.8 ^b^	58.2 ± 0.2 ^a^	25.1 ± 0.2 ^c^	5.5 ± 0.2 ^b^	42.4 ± 0.7 ^a^	5.6 ± 0.1 ^b^
quercetin	106.9 ± 6.7 ^c^	397.5 ± 1.8 ^a^	207.3 ± 8.6 ^b^	83.9 ± 0.6 ^b^	117.9 ± 1.8 ^a^	69.4 ± 1.0 ^c^
apigenin	103.4 ± 5.8 ^b^	133.4 ± 9.5 ^a^	116.7 ± 7.0 ^b^	90.4 ± 2.5 ^b^	26.7 ± 1.1 ^c^	99.4 ± 5.5 ^a^
kaempferol	25.5 ± 0.4 ^c^	96.8 ± 3.5 ^a^	36.4 ± 0.6 ^b^	-	-	-
myricetin	169.1 ± 4.8 ^c^	750.0 ± 9.8 ^a^	567.7 ± 7.2 ^b^	80.8 ± 4.0 ^b^	123.9 ± 2.5 ^a^	122.6 ± 0.7 ^a^
total	434.6 ± 4.7 ^c^	1435.9 ± 4.9 ^a^	953.2 ± 4.7 ^a^	260.7 ± 1.5 ^c^	310.9 ± 2.0 ^a^	297.0 ± 1.5 ^b^

Values are expressed as mean ± SD (*n* = 3). -: Not detected. Means with different letters are significantly different at *p* < 0.05 within the same row of each sample using different treatments.

**Table 3:** Proximate composition and oryzanol and tocopherols contents of stink bugs with different cooking methods.

Parameter	Late Nymph			Adult		
	Raw	Roasted	Grilled	Raw	Roasted	Grilled
Protein (g/100 g)	55.6 ± 0.4 ^a^	55.5 ± 0.5 ^a^	57.5 ± 1.3 ^a^	31.0 ± 0.6 ^b^	33.3 ± 0.1 ^a^	31.9 ± 0.1 ^b^
Fat (g/100 g)	23.5 ± 0.6 ^a^	23.4 ± 0.1 ^a^	23.0 ± 1.2 ^a^	50.5 ± 0.2 ^a^	50.7 ± 0.0 ^a^	51.4 ± 0.5 ^a^
Fiber (g/100 g)	14.2 ± 0.0 ^a^	12.7 ± 0.6 ^b^	12.5 ± 0.0 ^b^	14.3 ± 0.5 ^a^	12.1 ± 0.0 ^b^	11.7 ± 0.3 ^b^
Ash (g/100 g)	3.3 ± 0.1 ^b^	4.9 ± 0.0 ^a^	2.8 ± 0.0 ^c^	1.4 ± 0.1 ^b^	1.1 ± 0.0 ^b^	1.8 ± 0.1 ^a^
Carbohydrate (g/100 g)	3.4 ± 0.1 ^b^	3.5 ± 0.0 ^b^	4.1 ± 0.2 ^a^	2.8 ± 0.2 ^b^	2.9 ± 0.1 ^ab^	3.2 ± 0.0 ^a^
*δ*-tocopherol (μg/g)	0.5 ± 0.0 ^c^	0.7 ± 0.0 ^b^	1.4 ± 0.1 ^a^	2.7 ± 0.1 ^a^	2.3 ± 0.2 ^b^	0.7 ± 0.0 ^c^
*γ*-tocopherol (μg/g)	5.7 ± 0.0 ^b^	7.6 ± 0.2 ^a^	5.2 ± 0.3 ^c^	4.4 ± 0.3 ^b^	18.5 ± 0.7 ^a^	2.9 ± 0.2 ^c^
*α*-tocopherol (μg/g)	1.7 ± 0.0 ^c^	6.1 ± 0.1 ^a^	2.9 ± 0.2 ^b^	3.1 ± 0.1 ^b^	2.8 ± 0.1 ^c^	4.7 ± 0.3 ^a^
*γ*-oryzanol (mg/g)	-	-	-	-	-	-

Values are expressed as mean ± SD (*n* = 3). -: Not detected. Means with different letters are significantly different at *p* < 0.05 within the same row of each sample using different treatments.

**Table 4:** Amino acid content in stink bugs with different cooking methods (mg/g).

Parameter	Late Nymph			Adult		
	Raw	Roasted	Grilled	Raw	Roasted	Grilled
**Essential**						
arginine	13.6 ± 0.1 ^b^	14.2 ± 0.2 ^a^	13.8 ± 0.1 ^b^	13.8 ± 0.0 ^b^	14.9 ± 0.3 ^a^	13.5 ± 0.2 ^b^
histidine	24.9 ± 0.8 ^a^	24.2 ± 0.5 ^a^	23.9 ± 0.4 ^a^	20.4 ± 0.3 ^a^	19.2 ± 0.3 ^b^	18.8 ± 0.1 ^b^
isoleucine	13.3 ± 0.1 ^b^	13.9 ± 0.2 ^a^	14.0 ± 0.2 ^a^	13.3 ± 0.3 ^b^	14.3 ± 0.2 ^a^	13.2 ± 0.2 ^b^
leucine	11.7 ± 0.1 ^b^	12.4 ± 0.1 ^a^	12.4 ± 0.2 ^a^	11.7 ± 0.0 ^b^	12.7 ± 0.2 ^a^	11.6 ± 0.1 ^b^
lysine	4.5 ± 0.1 ^a^	4.0 ± 0.1 ^b^	4.0 ± 0.2 ^b^	4.2 ± 0.1 ^b^	4.3 ± 0.1 ^a^	3.8 ± 0.0 ^c^
methionine	53.0 ± 1.1 ^b^	54.0 ± 0.6 ^ab^	55.2 ± 0.5 ^a^	74.2 ± 2.5 ^b^	80.5 ± 1.4 ^a^	72.0 ± 1.9 ^b^
phenylalanine	10.7 ± 0.2 ^c^	11.0 ± 0.1 ^b^	11.3 ± 0.1 ^a^	10.9 ± 0.2 ^b^	12.0 ± 0.1 ^a^	10.7 ± 0.1 ^c^
threonine	5.9 ± 0.1 ^a^	6.0 ± 0.1 ^a^	5.9 ± 0.1 ^a^	5.4 ± 0.1 ^b^	5.8 ± 0.1 ^a^	4.9 ± 0.0 ^c^
tryptophan	-	-	-	-	-	-
valine	18.5 ± 0.1 ^c^	19.4 ± 0.0 ^a^	19.1 ± 0.0 ^b^	16.4 ± 0.1 ^a^	16.7 ± 0.3 ^a^	16.0 ± 0.1 ^b^
sum-essential	155.9 ± 0.6 ^b^	158.9 ± 1.2 ^a^	159.4 ± 0.7 ^a^	170.3 ± 2.5 ^b^	180.2 ± 1.4 ^a^	164.4 ± 2.0 ^c^
**Non-Essential**						
alanine	15.5 ± 0.2 ^a^	16.0 ± 0.1 ^a^	14.6 ± 0.7 ^b^	14.3 ± 0.1 ^a^	14.1 ± 0.1 ^a^	13.1 ± 0.1 ^b^
asparagine	-	-	-	-	-	-
aspartic acid	11.1 ± 0.1 ^a^	11.2 ± 0.2 ^a^	11.1 ± 0.2 ^a^	11.5 ± 0.1 ^b^	12.9 ± 0.2 ^a^	11.2 ± 0.2 ^c^
cysteine	0.1 ± 0.0 ^b^	0.1 ± 0.0 ^a^	0.1 ± 0.0 ^a^	0.1 ± 0.0 ^a^	0.1 ± 0.0 ^a^	0.1 ± 0.0 ^b^
glutamine	4.8 ± 0.1 ^a^	4.4 ± 0.1 ^b^	4.4 ± 0.0 ^b^	4.5 ± 0.1 ^b^	4.7 ± 0.1 ^a^	4.1 ± 0.1 ^c^
glutamic acid	13.0 ± 0.3 ^a^	12.7 ± 0.3 ^a^	12.9 ± 0.1 ^a^	12.9 ± 0.1 ^b^	14.4 ± 0.2 ^a^	12.3 ± 0.4 ^b^
glycine	4.0 ± 0.1 ^a^	3.8 ± 0.2 ^a^	3.7 ± 0.2 ^a^	4.2 ± 0.1 ^a^	4.1 ± 0.1 ^a^	3.7 ± 0.1 ^b^
proline	11.4 ± 0.8 ^a^	11.0 ± 0.4 ^ab^	10.1 ± 0.2 ^b^	11.2 ± 0.4 ^a^	11.3 ± 0.4 ^a^	10.6 ± 0.3 ^a^
serine	8.5 ± 0.2 ^ab^	8.7 ± 0.0 ^a^	8.3 ± 0.0 ^ab^	7.6 ± 0.1 ^a^	7.8 ± 0.1 ^a^	6.9 ± 0.1 ^b^
tyrosine	70.5 ± 0.8 ^b^	74.6 ± 0.1 ^a^	74.2 ± 0.6 ^a^	45.5 ± 0.3 ^a^	45.8 ± 0.6 ^a^	42.7 ± 0.1 ^b^
sum-non-essential	138.8 ± 0.7 ^b^	142.4 ± 1.1 ^a^	139.5 ± 0.8 ^b^	111.8 ± 0.5 ^b^	115.1 ± 1.0 ^a^	104.6 ± 0.3 ^c^
total amino acids	294.7 ± 0.8 ^b^	301.4 ± 1.7 ^a^	298.9 ± 1.4 ^a^	282.1 ± 2.3 ^b^	295.3 ± 2.2 ^a^	268.9 ± 1.9 ^c^
EAAI	0.48	0.49	0.49	0.42	0.43	0.40

Values are expressed as mean ± SD (*n* = 3). -: Not detected. Means with different letters are significantly different at *p* < 0.05 within the same row of each sample using different treatments.

**Table 5:** Volatile compounds content in stink bugs with different cooking methods.

No	Volatile Compounds ^a^	Ri ^b^	Late Nymph			Adult		
			Raw	Roasted	Grilled	Raw	Roasted	Grilled
1	Disulfide, dimethyl	741	0.10	0.21	0.28	0.62	0.74	0.26
2	Trisulfide, dimethyl	969	-	-	-	0.25	-	-
3	2-Hexenoic acid	1060	-	-	-	1.62	0.89	0.12
4	Undecane	1100	-	-	-	0.09	0.12	0.06
5	Butane,3-methyl-1-(methylthio)-	1137	-	-	-		0.07	
6	Dodecane	1200	1.57	1.91	1.46	2.86	3.15	2.48
7	2-Octenoic acid	1241	0.52	-	-	-	-	-
8	1-Tridecene	1293	0.28		0.27	0.36	0.33	0.33
9	Octadecane, 5-methyl-	1309	88.59	87.5	87.48	92.24	92.79	89.47
10	Pentadecane	1502	0.11	0.18	0.17	0.14	0.09	0.08
11	*cis*-9-Hexadecenoic acid	1957	-	-	0.49	-	-	-
12	Palmitoleic acid	1958	-	0.50	-	0.07	-	0.35
13	*cis*-10-Heptadecenoic acid	1960	0.20	-	-	-	0.05	-
14	*n*-Hexadecanoic acid	1976	2.19	3.24	3.24	0.70	0.68	3.36
15	*n*-Heptanoicacid,methyl (tetramethylene)silyl ester	1991	-	-	-	-	0.07	-
16	10(E),12(Z)-Conjugated linoleic acid	2149	0.76	0.78	0.94	-	-	-
17	Oleic Acid	2153	3.96	3.77	4.73	1.04	0.90	3.33
18	Octadecanoic acid	2177	1.28	1.50	0.24		0.12	
19	Heneicosane	2504	0.15	0.14	0.21	0.01	-	0.05
20	Cyclohexane,1,1′-tetradecylidenebis-	2567	-	-	-	-	-	0.08
21	9-Tricosanol, acetate	2690	0.14	0.13	0.25	-	-	-
22	Tetracontane	2706			0.24	-	-	-
23	Hexatriacontane	2707	0.15	0.14	-	-	-	0.03

-: Not detected. ^a^: Compounds listed in order of elution from NIST library; ^b^: Retention indices (RI) relative to *n*-alkanes (C7-C30) on elite-5MS capillary column.

**Table 6:** Half maximal inhibitory concentration (IC_50_) of the extracts from stink bugs compared to a positive control, Doxorubicin.

IC_50_ (µg/mL)	Doxorubicin	Extraction	Late Nymph			Adult		
			Raw	Roasted	Grilled	Raw	Roasted	Grilled
Hela	0.06 ± 0.01	cold extraction *	>2000	>2000	>2000	>2000	>2000	>2000
		heat extraction **	747.5 ± 21.5	394.9 ± 30.0	715.8 ± 52.1	625.2 ± 26.8	585.2 ± 25.9	257.7 ± 21.9
KB	0.26 ± 0.01	cold extraction	>2000	>2000	>2000	>2000	>2000	>2000
		heat extraction	592.2 ± 25.3	285.4 ± 5.2	298.0 ± 6.8	558.4 ± 28.9	395.0 ± 25.7	303.1 ± 16.0
Vero	1.91 ± 0.39	cold extraction	>2000	>2000	>2000	>2000	>2000	>2000
		heat extraction	1282.1 ± 10.3	670.7 ± 35.3	737.4 ± 35.8	1279.0 ± 5.8	639.8 ± 14.2	647.2 ± 17.6

Hela–Human cervical carcinoma cell line, KB–Human oral epidermoid carcinoma cell line, Vero–African green monkey kidney cell line. * extracted at room temperature, ** extracted at 90 °C in a water bath.

## Data Availability

Data is contained within the article and supplementary material.

## Supplementary Materials

The following supporting information can be downloaded at: https://www.mdpi.com/article/10.3390/foods12163053/s1, Table S1: Linear equations for the quantitative calculation of concentration.

## References

[1] Kinyuru J.N., Mogendi J.B., Riwa C.A., Ndung’u N.W. (2015). Edible insects—A novel source of essential nutrients for human diet: Learning from traditional knowledge. Anim. Front..

[2] Wu R.A., Ding Q., Yin L., Chi X., Sun N., He R., Luo L., Ma H., Li Z. (2020). Comparison of the nutritional value of mysore thorn borer (*Anoplophora chinensis*) and mealworm larva (*Tenebrio molitor*): Amino acid, fatty acid, and element profiles. Food Chem..

[3] Fogang Mba A.R., Kansci G., Viau M., Hafnaoui N., Meynier A., Demmano G., Genot C. (2017). Lipid and amino acid profiles support the potential of *Rhynchophorus phoenicis* larvae for human nutrition. J. Food Compos. Anal..

[4] Yi L., Lakemond C.M.M., Sagis L.M.C., Eisner-Schadler V., Van Huis A., Van Boekel M.A.J.S. (2013). Extraction and characterisation of protein fractions from five insect species. Food Chem..

[5] Köhler R., Kariuki L., Lambert C., Biesalski H.K. (2019). Protein, amino acid and mineral composition of some edible insects from Thailand. J. Asia-Pac. Entomol..

[6] Raksakantong P., Meeso N., Kubola J., Siriamornpun S. (2010). Fatty acids and proximate composition of eight Thai edible terricolous insects. Food Res. Int..

[7] Ordoez-Araque R., Egas-Montenegro E. (2021). Edible insects: A food alternative for the sustainable development of the planet. Int. J. Gastron. Food Sci..

[8] Aiello D., Barbera M., Bongiorno D., Cammarata M., Censi V., Indelicato S., Mazzotti F., Napoli A., Piazzese D., Saiano F. (2023). Edible insects an alternative nutritional source of bioactive compounds: A review. Molecules.

[9] da Silva Lucas A.J., de Oliveira L.M., da Rocha M., Prentice C. (2020). Edible insects: An alternative of nutritional, functional and bioactive compounds. Food Chem..

[10] Wang Y., Zhao D., Gao J., Peng Z. (2015). Determination of the volatile composition of *Tessaratoma papillosa* NYMPH by GC/MS. Chem. Nat. Compd..

[11] Hlongwane Z.T., Siwela M., Slotow R., Munyai T.C. (2022). Effect of geographical location, insect type and cooking method on the nutritional composition of insects consumed in South Africa. J. Insects Food Feed.

[12] Ssepuuya G., Nakimbugwe D., De Winne A., Smets R., Claes J., Van Der Borght M. (2020). Effect of heat processing on the nutrient composition, colour, and volatile odour compounds of the long-horned grasshopper *Ruspolia differens* serville. Food Res. Int..

[13] Chumroenphat T., Somboonwatthanakul I., Saensouk S., Siriamornpun S. (2021). Changes in curcuminoids and chemical components of turmeric (*Curcuma longa* L.) under freeze-drying and low-temperature drying methods. Food Chem..

[14] Kubola J., Siriamornpun S., Meeso N. (2011). Phytochemicals, vitamin C and sugar content of Thai wild fruits. Food Chem..

[15] Chen M., Bergman C.J. (2005). A rapid procedure for analysing rice bran tocopherol, tocotrienol and *γ*-oryzanol contents. J. Food Compos. Anal..

[16] Zhang Z., Wu W., Li G. (2009). Study of the alarming volatile characteristics of *Tessaratoma papillosa* using SPME-GC-MS. J. Chromatogr. Sci..

[17] Siripong P., Rassamee K., Piyaviriyakul S., Yahuafai J., Kanokmedhakul K. (2012). Anti-metastatic effects on B16F10 melanoma cells of extracts and two prenylated xanthones isolated from *Maclura amboinensis* Bl roots. Asian Pac. J. Cancer Prev..

[18] Koch R.L., Pezzini D.T., Michel A.P., Hunt T.E. (2017). Identification, biology, impacts, and management of stink bugs (Hemiptera: Heteroptera: Pentatomidae) of soybean and corn in the midwestern United States. J. Integr. Pest Manag..

[19] Sanders D., Nickel H., Grützner T., Platner C. (2008). Habitat structure mediates top–down effects of spiders and ants on herbivores. Basic Appl. Ecol..

[20] Hassan A.B., Al Maiman S.A., Alshammari G.M., Mohammed M.A., Alhuthayli H.F., Ahmed I.A.M., Alfawaz M.A., Yagoub A.E.A., Fickak A., Osman M.A. (2021). Effects of boiling and roasting treatments on the content of total phenolics and flavonoids and the antioxidant activity of peanut (*Arachis hypogaea* L.) pod shells. Processes.

[21] Kamalaja T., Prashanthi M., Rajeswari K. (2018). Evaluation of antioxidant activity and bioactive compounds on domestic cooking method. Int. J. Curr. Microbiol. Appl. Sci..

[22] Chukwumah Y., Walker L., Vogler B., Verghese M. (2007). Changes in the phytochemical composition and profile of raw, boiled, and roasted peanuts. J. Agric. Food Chem..

[23] Mohamed Ahmed I.A., Al Juhaimi F.Y., Osman M.A., Al Maiman S.A., Hassan A.B., Alqah H.A.S., Babiker E.E., Ghafoor K. (2020). Effect of oven roasting treatment on the antioxidant activity, phenolic compounds, fatty acids, minerals, and protein profile of Samh (*Mesembryanthemum forsskalei* Hochst) seeds. LWT.

[24] Adisakwattana S. (2017). Cinnamic acid and its derivatives: Mechanisms for prevention and management of diabetes and its complications. Nutrients.

[25] Jannat B., Oveisi M.R., Sadeghi N., Hajimahmoodi M., Behzad M., Nahavandi B., Tehrani S., Sadeghi F., Oveisi M. (2013). Effect of roasting process on total phenolic compounds and *γ*-tocopherol contents of Iranian sesame seeds (*Sesamum indicum*). Iran. J. Pharm. Res..

[26] Silva M.P., Martinez M.J., Casini C., Grosso N.R. (2010). Tocopherol content, peroxide value and sensory attributes in roasted peanuts during storage. Int. J. Food Sci. Technol..

[27] Oluwaniyi O.O., Dosumu O.O., Awolola G.V. (2016). Effect of cooking method on the proximate, amino acid and fatty acid compositions of *Clarias gariepinus* and *Oreochromis niloticus*. J. Turk. Chem. Soc. A.

[28] Markmanuel D.P., Godwin J. (2020). Effects of culinary methods on the proximate composition of an edible insect (*Rhynchophorus Phoenicis*) larvae obtained from Bayelsa State, Nigeria. Eur. J. Agric. Food Sci..

[29] Thomas S., Vásquez-Benítez J.D., Cuéllar-Cepeda F.A., Mosquera-Vásquez T., Narváez-Cuenca C.E. (2021). Vitamin C, protein, and dietary fibre contents as affected by genotype, agro-climatic conditions, and cooking method on tubers of *Solanum tuberosum* Group Phureja. Food Chem..

[30] Cano-Estrada A., Castañeda-Ovando A., Ramírez-Godinez J., Contreras-López E. (2018). Proximate and fatty acid composition in raw and cooked muscle tissue of farmed rainbow trout (*Oncorhynchus mykiss*) fed with commercial fishmeal. J. Food Process. Preserv..

[31] Adu O.B., Ogundeko T.O., Ogunrinola O.O., Saibu G.M., Elemo B.O. (2015). The effect of thermal processing on protein quality and free amino acid profile of *Terminalia catappa* (Indian almond) seed. J. Food Sci. Technol..

[32] Lopes A.F., Alfaia C.M.M., Partidário A.M.C.P.C., Lemos J.P.C., Prates J.A.M. (2015). Influence of household cooking methods on amino acids and minerals of Barrosã-PDO veal. Meat Sci..

[33] Ashraf I., Zubair M., Rizwan K., Rasool N., Jamil M., Khan S.A., Tareen R.B., Ahmad V.U., Mahmood A., Riaz M. (2018). Chemical composition, antioxidant and antimicrobial potential of essential oils from different parts of *Daphne mucronata* Royle. Chem. Cent. J..

[34] Domínguez R., Gómez M., Fonseca S., Lorenzo J.M. (2014). Effect of different cooking methods on lipid oxidation and formation of volatile compounds in foal meat. Meat Sci..

[35] Van H., Hwang I., Jeong D., Touseef A., Akyar I. (2012). Principle of meat aroma flavors and future prospect. Latest Research into Quality Control.

[36] Yu P. (2005). Protein secondary structures (*α*-helix and *β*-sheet) at a cellular level and protein fractions in relation to rumen degradation behaviours of protein: A new approach. Br. J. Nutr..

[37] Waszkowiak K., Mikołajczak B. (2020). The effect of roasting on the protein profile and antiradical capacity of flaxseed meal. Foods.

[38] Manditsera F.A., Luning P.A., Fogliano V., Lakemond C.M.M. (2019). Effect of domestic cooking methods on protein digestibility and mineral bioaccessibility of wild harvested adult edible insects. Food Res. Int..

