# Supplementary material for: Evaluation of Roasting and Grilling Effects on Chemical Composition, Volatile Profiles, and Toxicity of Stink Bugs (Tessaratoma papillosa): Implications for Utilization as Functional Food Ingredients
Source: Foods. 2023 Aug 15;12(16):3053. doi: 10.3390/foods12163053 (PMC10453842; doi:10.3390/foods12163053)
Supplement: Supplementary file 1 [file foods-12-03053-s001.zip › foods-2554651-supplementary.pdf]

Table S1. Linear equations for the quantitative calculation of concentration.

Parameter	Standard equation	R^2
**Phenolic acid**		
gallic acid	y = 78019x − 1274.3	0.9998
protocatechuic acid	y = 43642x + 8207.3	0.9999
p-hydroxybenzoic acid	y = 78883x − 73853	0.9998
chlorogenic acid	y = 38055x − 15157	0.9998
vanillic acid	y = 49696x − 2158.4	0.9998
caffeic acid	y = 156014x − 8841.6	0.9993
syringic acid	y = 72068x − 802.83	0.9999
vanillin	y = 119664x + 207.42	0.9996
p–coumaric acid	y = 175186x − 286.17	0.9999
ferulic acid	y = 150983x − 2196.5	0.9999
sinapic acid	y = 140735x − 634.42	0.9999
cinamic acid	y = 103334x − 645.4	0.9999
genistic acid	y = 47645x + 1893	0.9998
**Flavonoid**		
rutin	y = 26626x − 11367	0.9996
quercetin	y = 51540x − 77311	0.9995
apigenin	y = 23380x − 14228	0.9998
kaempferol	y = 22910x − 22300	0.9987
myricetin	y = 51540x − 77311	0.9988
δ-tocopherol	y = 5998.3x − 477.78	0.9999
γ-tocopherol	y = 3991.8x + 258.61	0.9999
α-tocopherol	y = 3388.9x + 15.781	0.9999
γ-oryzanol	y = 33624x – 24829	0.9999
**Amino acid**		
arginine	y = 1,326,233.78x − 951,905.91	0.9957
histidine	y = 2,149,993.78x − 1,111,311.29	0.9982
isoleucine	y = 11,571,543.08x + 1,590,415.08	0.9999
leucine	y = 13,690,663.96x + 578,577.49	0.9999
lysine	y = 1,791,508.92x − 13,558.21	0.9996
methionine	y = 708728x − 2986.8	0.9996
phenylalanine	y = 2,868,939.29x − 3,688.08	0.9999
threonine	y = 583437x − 11823	0.9996
tryptophan	y = 1,523,896.10x − 11,738.72	0.9998
valine	y = 5,201,361.51x − 55,119.67	0.9999
alanine	y = 1,408,972.21x − 4,255.64	0.9999
asparagine	y = 437380x − 16155	0.9999
aspartic acid	y = 574160x − 1580	0.9997
cysteine	y = 257,138.01x − 13,444.25	0.9998
glutamine	y = 2,088,132.64x − 20,309.93	0.9999
glutamic acid	y = 917690x − 12460	0.9987
glycine	y = 287,042.77x − 2,292.34	0.9985
proline	y = 5,392,531.38x − 27,271.73	0.9999
serine	y = 1,093,065.34x − 50,688.57	0.9999
tyrosine	y = 438730x − 3568.60	0.9997

The concentration ranges of phenolic acids, flavonoids, γ-oryzanol, and tocopherols were all 2.00–1000 μg/mL, and that of amino acids was 0.10–50 μg/mL.
